# Phenotypic characterisation of bovine alveolar macrophages reveals two major subsets with differential expression of CD163

**DOI:** 10.1038/s41598-024-65868-7

**Published:** 2024-06-28

**Authors:** Emily M. Randall, Paul Sopp, Anna Raper, Inga Dry, Tom Burdon, Jayne C. Hope, Lindsey A. Waddell

**Affiliations:** 1 The Roslin Institute, Easter Bush, Edinburgh, EH25 9RG UK; 2 MRC Weatherall Institute for Molecular Medicine, John Radcliffe Hospital, Oxford, OX39DS UK

**Keywords:** Monocytes and macrophages, Flow cytometry

## Abstract

Bovine alveolar macrophages (AMs) defend the lungs against pathogens such as *Mycobacterium bovis* (*M. bovis)*, the causative agent of bovine tuberculosis. However, little is known about the surface molecules expressed by bovine AMs and whether there is heterogeneity within the population. The purpose of this study was to characterise the bovine AM cell surface phenotype using flow cytometry. Bronchoalveolar lavage samples from four different calves were stained with a combination of antibodies against immune cell molecules prior to flow cytometric analysis. To assess the degree of expression, we considered the distribution and relative intensities of stained and unstained cells. We demonstrated that bovine AMs have high expression of CD172a, ADGRE1, CD206, and CD14, moderate expression of CD80, MHC II, CD1b, and CD40, low expression of CX3CR1 and CD86, and little or no expression of CD16 and CD26. Two distinct subsets of bovine AMs were identified based on CD163 expression. Subsequent analysis showed that the CD163^+^ subset had greater expression of other typical macrophage molecules compared to the CD163^-^ subset, suggesting that these cells may perform different roles during infection. The characterisation of the uninfected bovine AM phenotype will provide a foundation for the examination of *M. bovis-*infected AMs.

## Introduction

*Mycobacterium bovis* is the pathogen responsible for bovine tuberculosis, a significant global disease which impacts both animal and human health. Alveolar macrophages (AMs) are the first line of defence against *M. bovis* and the early interactions between host and pathogen are crucial in determining the outcome of infection^1^. Despite this, little is known about the phenotypic diversity of bovine AMs, including the cell surface molecules present.

Knowledge of the bovine AM phenotype is essential for understanding infections that target this immune cell and may provide important insights for disease control. We previously used single colour flow cytometry to describe bovine AMs^2^, and here we significantly extend these findings using multi-colour analyses to include detection of additional molecules for which we have recently generated new reagents. This includes ADGRE1, the large animal homologue of murine F4/80; a G protein-coupled receptor (GPCR) with seven transmembrane domains and an extracellular region consisting of EGF-like repeats^3^. In mice, F4/80, encoded by the *Adgre1* gene, has been used as a marker for tissue-resident macrophages, although this does not include murine AMs which express low levels of F4/80^4,5^. The AMs of other animal species, however, have shown high levels of ADGRE1. In the pig, AMs express ADGRE1 at the cell surface, and ADGRE1 was hypothesised to function as a pattern recognition receptor. Based on RNA-Seq data, the expression of ADGRE1 in human, sheep, and buffalo AMs varies between the different species, but the degree of expression in bovine AMs was not measured^6^.

Another seven transmembrane GPCR found on the cell surface is the fractalkine receptor, CX3CR1, which regulates macrophage function at inflammatory sites^7^. Like mouse and human monocytes, bovine non-classical monocytes have high CX3CR1 gene expression which likely aids migration into inflamed tissues, whereas classical monocytes have low expression^8,9^. In the murine lung, interstitial macrophages are CX3CR1 positive whilst AMs are CX3CR1 negative^10^. Detection of CX3CR1 expression on the surface of bovine AMs through use of the fluorescently labelled single ligand, CX3CL1, as a molecular tag for receptor expression enables further studies of this molecule.

The mannose receptor, CD206, has been used as a marker for the M2, anti-inflammatory macrophage state. Both human and murine AMs express high levels of CD206 in the normal, healthy lung^11,12^. Human AMs also variably express the M2 related molecule CD163. These macrophages can be divided into high CD163 and intermediate CD163 subpopulations. It has been speculated that the heterogeneity in CD163 expression may be due to a mix of tissue-resident and monocyte-derived macrophages in the population, or to different anatomical positions or activation states^11^.

The heterogeneity of the AM population becomes more pronounced when macrophages are exposed to pathogen associated molecular patterns. Activated macrophages upregulate pro-inflammatory genes and those involved in antigen presentation. This includes MHC class II and CD1b which present peptide and lipid antigens respectively to T-cells. The co-stimulatory molecules, CD80 and CD86, are also upregulated as they are necessary for T-cell activation. These pro-inflammatory M1 macrophages also upregulate CD40 which enhances their activity via interaction with CD40L on T-cells^13^. It is likely that the characteristics, diversity, and abundance of AM populations change over time depending upon the microenvironment. In the murine lung, M2 macrophage subpopulations dominate prior to inflammation. The induction of inflammation leads to an increase in M1 macrophage subpopulations which subsequently almost disappear during the resolution of inflammation^14^. Understanding how AM populations change in cattle during *M. bovis* infection could provide significant insight into the early host–pathogen interactions that control the outcome of exposure and pinpoint targets for intervention.

Few studies have analysed bovine AMs by flow cytometry. However, it has been shown that the majority of cells in broncho-alveolar lavage (BAL) fluid from healthy calves are positive for expression of both the phagocytosis inhibitor, CD172a, and the LPS receptor, CD14. Conversely, very few of these cells expressed the Fc receptor, CD16^2^. These findings differ from those of human studies which have shown that human AMs have low CD14 expression and are positive for CD16^15^. This study aimed to characterise bovine AMs further using multicolour flow cytometry panels to detect the presence of various macrophage markers. Using novel reagents, we show uniform expression of ADGRE1 on bovine BAL cells, low expression of CX3CR1, and describe two major subsets of macrophages with differential expression of CD163.

## Materials and methods

### Calves and sample collection

The calves used in this study and the collection of BAL fluid has been previously described^2^. All samples were taken with ethical approval from the Veterinary Ethics and Review Committee at the Royal (Dick) School of Veterinary Studies in line with the Animal Research: Reporting of In Vivo Experiments (ARRIVE) Guidelines^16^. Bronchoalveolar lavage was performed on cadavers and is not classified as a regulated procedure under the Animals (Scientific Procedures) Act, 1986 which governs animal studies in the UK. Briefly, BAL samples were taken from Holstein–Friesian male calves aged between 12 and 24 days. The calves were humanely euthanised by captive bolt and death was confirmed by auscultation. The lungs were excised with the trachea intact and subsequently, lavage was performed by pouring 1 L of sterile phosphate buffered saline (PBS) into the lungs via a funnel inserted into the trachea. Lungs were massaged for 1 min before decanting the BAL fluid into a container. The BAL fluid was processed further by filtering through a 70 µm filter, and cells were washed and counted as previously described^17^. Cells were cryopreserved in Foetal Calf Serum (Thermo Fisher Scientific; USA) containing 10% DMSO (Sigma-Aldrich; USA) and stored at − 155 °C prior to characterisation by flow cytometry.

### Construction and production of ADGRE1 immunogens

The cDNA of the N-terminal extracellular EGF-like domains for bovine (ENSBTAT00000010390.6, e!Ensembl), ovine (ENSOART00000005245.1, e!Ensembl), human (ENST00000312053.8, e!Ensembl) and rat (NM_001007557.1, NCBI) were synthesized by Synbio Technologies and subsequently subcloned in frame into pFUSE–hIgG1-Fc2 vector (Invivogen; San Diego, USA) using EcoRI-BglII site for bovine, ovine and human sequences and EcoRV-BglII site for the rat sequence. Recombinant protein production was carried out as previously described^6^.

### Generation of anti-bovine ADGRE1 monoclonal antibody (mAb)

This work was carried out under the authority of a UK Home Office Project License under the regulations of the Animals (Scientific Procedures) Act 1986, with approval from the Roslin Institute Animal Welfare and Ethics Committee. Balb/c mice (Charles River Laboratories, UK) received three subcutaneous immunisations, 21 days apart comprising 50 μg protein (12.5 μg each of ovine, bovine, rat ADGRE1 and human EMR1 protein in PBS) with TiterMax Gold adjuvant (Merck, UK) in maximum volume of 100 μl. A final intraperitoneal injection with 50 μg protein (as above) in PBS was provided 4 days prior to cull. Cells were flushed from the spleens with RPMI1640 supplemented with Glutamax (both Gibco, USA), and were fused with Sp2/0-Ag14 mouse myeloma cells (CRL-1581; ATCC) at a ratio of 5:1, as previously described^18^.

An indirect ELISA, as previously described^18^, against recombinant ovine, bovine, rat ADGRE1 and human EMR1 Fc fusion protein (all at 50 ng per well) was used to identify hybridomas which were producing specific antibodies. Recombinant human IgG Fc protein was used to discriminate non-specific reactivity to the human-IgG1-Fc fusion tag. Positive hybridoma cells were expanded and subcloned by serial dilution^18^. Supernatants from expanded clones were purified by Protein G HiTrap column (Merck, UK), with buffer exchange carried out using Slide-A-Lyzer™ G2 Dialysis Cassettes 1–3 mL 10 K MWCO (Thermo Fisher Scientific, UK). The isotype was determined using the IsoStrip mouse monoclonal antibody isotyping kit (Roche, UK).

### Generation of bovine CX3CL1

The DNA sequence encoding bovine CX3CL1 was flanked with EcoRI (GAATTC) and BglII (AGATCT) restriction sites. The designed DNA fragment was synthesized by Synbio Technologies (Monmouth Junction, USA) and was subsequently cloned into the vector pFUSE-hIgG1-FC1 (Invivogen; San Diego, USA). The restriction enzymes EcoRI and BglII were both supplied by New England Biolabs (Hitchin, UK). The pFUSE-CX3CL1-FC1 was transformed into chemically competent Escherichia coli DH5α, using a standard heat-shock protocol. Transformed bacteria were recovered for 1 h in LB media at 37 °C, with shaking at 180 rpm, prior to plating on LB media agar supplemented with 25 μg/ml Zeocin (ant-zn-05; Invivogen; San Diego, USA). All further growth of the transformed bacteria occurred in LB media supplemented with 25 μg/ml Zeocin. Sanger sequencing (Eurofinsgenomics; Ebersberg, Germany) was used to confirm correct insertion and sequence of the CX3CL1 in the final expression vector. DNA was prepared for transfection, in accordance with the manufacturer’s instructions, using an Endofree Maxiprep kit (Qiagen; Manchester, UK) and the DNA was resuspended in 1 × TE buffer prior to quantification by a Nanodrop Spectrophotometer (Thermo Fisher Scientific; Perth, UK). Mycoplasma free HEK293T cells used for transfection were cultured prior to use in DMEM (Merck; Darmstadt, Germany), supplemented with 8% Ultra Low IgG foetal bovine serum (Thermo Fisher Scientific; Perth, UK) and Glutamax (Thermo Fisher Scientific; Perth, UK) at 37 °C/5% CO_2_. For each flask used, 90 μg of DNA was complexed with 90 μl of Lipofectamine 2000 (Thermo Fisher Scientific; Perth, UK) in Opti-MEM reduced serum media (Thermo Fisher Scientific; Perth, UK) for 10 min at room temperature. Transfections were allowed to proceed for 5 days before supernatants were harvested. Supernatants were cleared by centrifugation at 375 × g and filtered through a 0.45 μM and 0.22 μM low protein binding filter (Millipore; Livingston, UK) prior to application to a 1 ml HiTrap protein G HP column (HP 17-0404-01; Cytivia; Little Chalfont, UK) which had been stripped with 5 × column volumes of 0.1 M Glycine pH 2.6 and washed/equilibrated with 10 × column volumes of PBS prior to use. For all steps a flow rate of 1 ml/min was used. Following binding of the expressed protein to the protein G, the column was washed with 10 × column volumes of PBS. Bound protein was eluted from the column in 1 ml fractions, using 0.1 M Glycine pH 2.7. Each fraction collected was neutralized by the addition of 50 μl of 1 M Tris pH 9.0. Fractions containing protein were identified using a Nanodrop Spectrophotometer (Thermo Fisher Scientific; Perth, UK), pooled and then buffer exchanged into sterile PBS using a 30 kDa MW cut-off Amicon Ultra-4 centrifugation filter unit (Merck; Darmstadt, Germany). The final concentration of CXC3L1 was determined using a Nanodrop Spectrophotometer (Thermo Fisher Scientific; Perth, UK) and fluorescently labelled using Molecular Probes Alexa Fluor 647 conjugation kit (Thermo Fisher Scientific; USA) according to manufacturer’s instructions.

### Flow cytometry

Primary monoclonal antibodies (mAbs) were conjugated to fluorophores using either the Molecular Probe kit (Thermo Fisher Scientific; USA) for Pacific Blue, Alexa Fluor 488 (AF488), Alexa Fluor 568 (AF568) and Alexa Fluor 647 (AF647), or the Lightning Link kit (Abcam; UK) for PE-Cy5, PE-Cy7, and PerCP-Cy5.5. Each conjugated reagent was titrated to determine an optimal dilution (Table 1).

**Table 1:** Antibodies used for multicolour flow cytometry analysis.

Molecule	Clone	Species	Conjugate	Isotype	Optimal dilution	Source	Secondary antibody
CD172a	ILA24	Mouse anti-bovine	Pacific Blue	IgG1	1:100	RI Toolbox	N/A
ADGRE1	1F6/1A6	Mouse anti-bovine	AF488	IgG1	1:500	RI Toolbox	N/A
CD163	LND68A	Mouse anti-bovine	PerCP-Cy5.5	IgG1	1:2000	Kingfisher, USA	N/A
CD26	CC69	Mouse anti-bovine	AF568	IgG1	1:100	BioRad, USA	N/A
CD206	122D2.08	Mouse anti-human	None	IgG1	1:2500 (0.2 ug/ml)	Dendritics, France	Rat anti-mouse IgG1 Clone: RMG1-1 Conjugate: PE-Cy7 Conc.: 0.01ug/ml Source: BioLegend, USA
CX3CR1			AF647		1:50	RI Toolbox	N/A
MHC II	ILA21	Mouse anti-bovine	AF488	IgG2a	1:200	RI Toolbox	N/A
CD86	IL-A190	Mouse anti-bovine	PerCP-Cy5.5	IgG1	1:200	RI Toolbox	N/A
CD80	IL-A159	Mouse anti-bovine	AF568	IgG1	1:200	RI Toolbox	N/A
CD1b	CC122	Mouse anti-bovine	None	IgG1	1:1000	RI Toolbox	Rat anti-mouse IgG1 Clone: RMG1-1 Conjugate: PE-Cy7 Conc.: 0.01ug/ml Source: BioLegend, USA
CD40	IL-A158	Mouse anti-bovine	AF647	IgG1	1:400	RI Toolbox	N/A
CD16	KD1	Mouse anti-human	FITC	IgG2a	1:200 (5ug/ml)	BioRad, USA	N/A
CD14	CC-G33	Mouse anti-bovine	AF568	IgG1	1:200	BioRad, USA	N/A

RI Toolbox: Roslin Institute Immunological Toolbox; N/A not applicable.

Cryopreserved cells were thawed and washed in PBS, and the number of viable cells calculated using a haemocytometer and Trypan blue (Sigma-Aldrich; USA). Cells were resuspended in blocking buffer (PBS with 5% normal goat serum) to give 1 × 10^6^ cells per well. Cells were incubated with primary mAbs, diluted in blocking buffer for 30–60 min on ice, followed by three PBS washes. Secondary antibody was diluted in PBS before being added as indicated and incubated on ice for 30–60 min. This was followed by a further three PBS washes, and the addition of Zombie NIR viability dye (Biolegend; USA) at a 1:2000 dilution in PBS. After 20 min incubation at room temperature, cells were washed twice in PBS and flow cytometric analysis carried out using a LSRFortessa flow cytometer (BD Biosciences; USA). A minimum of 50,000 live, single cells were recorded for each sample.

The exact concentrations of the in-house conjugated antibodies were unknown; therefore, it was not possible to include concentration-matched isotype controls. However, before the cells were stained with the panels, flow cytometric analysis was carried out on unconjugated versions of the antibodies with the inclusion of concentration-matched isotype controls. This confirmed the absence of any non-specific binding (data not shown).

### Data analysis

All flow cytometry data was analysed using FlowJo_v10 software (BD Biosciences; USA). Compensation was first calculated and applied to all samples. Gates were drawn to exclude debris, doublets and select for viable cells (Supplementary Fig. 1). FMO controls were included in all panels to enable gates to be drawn which separated the positive and negative populations. Quadrants were applied to bivariate dot-plots of fluorescence data to determine whether the cells were single positive, double positive, or double negative (Supplementary Fig. 2). Microsoft Excel was used to produce all tables.

### Statistical analysis

Differences between expression of the surface molecules on the CD163^+^ and CD163^-^ subsets of AMs were assessed using a two-tailed paired Students’ T-test in Excel. *p* values of < 0.05 were considered significant.

## Results

### Bovine AMs express high levels of CD172a, ADGRE1, CD206 and CD14, and lower levels of CD80, MHC II, CD1b, CD40 and CX3CR1

The aim of this study was to characterise the AM populations in calf BAL fluid using multicolour flow cytometry to detect the presence of various cell surface expressed molecules.

**Figure 1:**
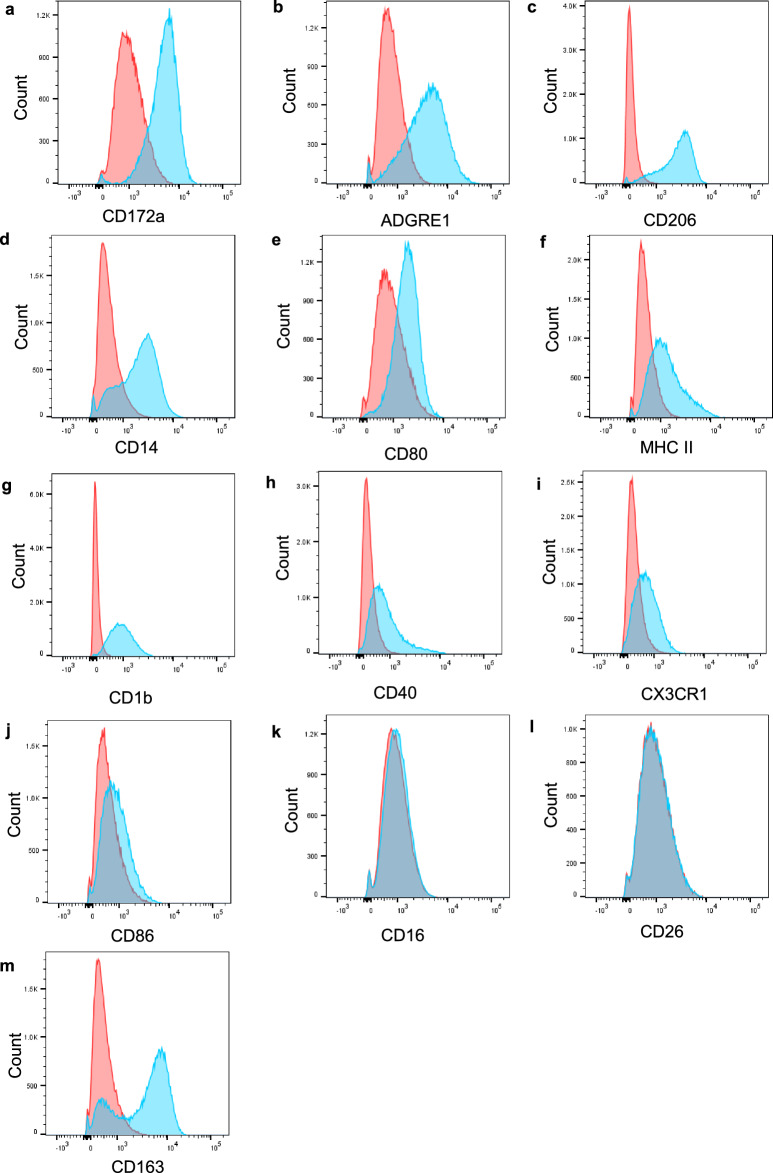
Expression of cell surface molecules by BAL Cells. Bovine BAL cells were stained for expression of the cell surface molecules shown and assessed by flow cytometry. Live, single cells were gated as described in Supplementary Figs. 1 and 2. Expression levels of each molecule are shown in blue histograms with unstained cells shown in red. One representative example of n = 4 biological replicates.

All four BAL samples were shown to have high autofluorescence and this prevented clear distinction of positive cells from negative cells, making it difficult to accurately calculate the percentage of cells which were positive for each molecule. Although the quadrants based on the FMO controls may underestimate the true proportion of positive cells (Supplementary Fig. 2), we assessed the degree of cell surface marker expression by calculating the change in median fluorescent intensity (MFI) values (Table 2), and considering the position of the histogram peaks of stained compared to unstained cells (Fig. 1).

**Table 2:** Mean (± SD) change in MFI for cell surface expressed molecules comparing unstained and stained cells.

	Mean change in MFI	SD
CD172a	3278.3	1806.6
ADGRE1	3462.8	1098.4
CD206	2706.0	694.4
CD14	1189.5	393.6
CD80	1069.5	118.3
MHCII	790.0	88.8
CD1b	742.5	152.2
CD40	614.3	301.2
CX3CR1	397.0	97.5
CD86	292.3	19.4
CD16	178.8	123.2
CD26	36.0	23.8

n = 4 biological replicates.

Three of the four BAL samples expressed high levels of CD172a (Fig. 1a). The BAL samples showed consistently high proportions of cells expressing ADGRE1 (Fig. 1b), CD206 (Fig. 1c), and CD14 (Fig. 1d). The expression of these molecules showed mean changes in MFI above 1100 (Table 2), and had large histogram peak shifts between the unstained and stained cells. The shifts in histogram peaks were more modest for CD80 (Fig. 1e), MHC II (Fig. 1f), CD1b (Fig. 1g), and CD40 (Fig. 1h), with mean changes in MFI between 500 and 1100 (Table 2). These molecules were therefore classed as moderately expressed. There were low levels of CX3CR1 (Fig. 1i) and CD86 (Fig. 1j) which showed minimal shifts in the histogram peaks, and had mean changes in MFI below 500 (Table 2). The majority of cells appeared to be positive for CX3CR1 however, despite the low MFI. Little or no expression of CD16 (Fig. 1k) and CD26 (Fig. 1l) was evident. In contrast to the above molecules which did not appear to be expressed on subsets of BAL cells, there were two clear histogram peaks of CD163 (Fig. 1m) in the BAL samples, indicating the presence of distinct CD163^+^ and CD163^-^ subsets.

### Subsets of bovine AMs differentially express CD163

**Figure 2:**
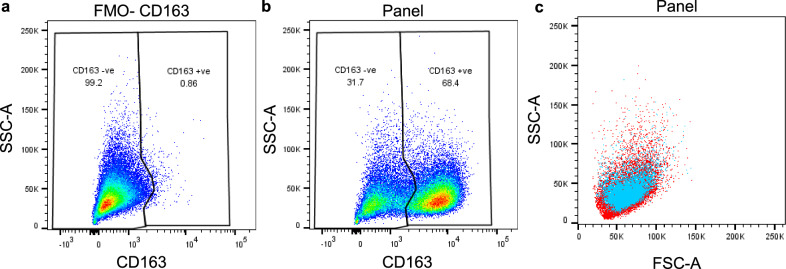
Subsets of BAL differentially express CD163. BAL cells were stained for expression of a range of cell surface molecules and assessed by flow cytometry. Live, single cells were gated as shown in Supplementary Fig. 1. (**a**) The FMO-CD163 control was used to draw a gate to separate cells into CD163^+^ and CD163^-^ subsets. (**b**) These gates were applied to the stained samples. (**c**) back-gating onto the FSC-A vs SSC-A plot showed that the CD163^−^ cells (red) and CD163^+^ cells (blue) were of a similar size. One representative example of n = 4 biological replicates.

**Figure 3:**
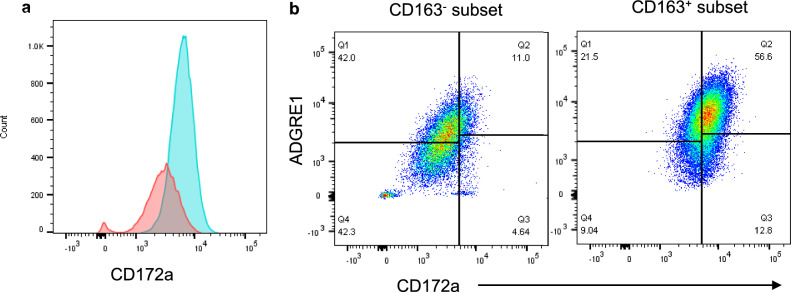
Differential expression of CD172a by CD163^+^ and CD163^−^ BAL cells. BAL cells were stained for expression of a range of cell surface molecules and assessed by flow cytometry. Live, single cells were gated as shown in Supplementary Fig. 1 and gates were set as in Supplementary Fig. 2. Cells were further gated based on expression of CD163 as shown in Fig. 2. (**a**) CD172a fluorescence intensities for the CD163^−^ subset (red) and the CD163^+^ subset (blue). (**b**) The cells were separated into CD163^+^ and CD163^-^ subsets before applying the CD172a vs ADGRE1 gate. One representative example of n = 4 biological replicates.

Cells were gated into CD163^+^ and CD163^-^ subpopulations for further analysis (Fig. 2) and the mean change in MFI for cell surface expressed molecules within each subset calculated (Table 3). The majority of cells were CD163^+^ (Fig. 2b; *Mean* = 66.2%, *SD* = 5.3%). In all BAL samples, the CD163^+^ population had significantly higher levels of CD172a, ADGRE1, CD206, CX3CR1, CD14, MHC II, CD26, and CD16 in comparison to the CD163^-^ cells. These differences appear not to be due to the intrinsic cell properties because the light scattering levels from both the CD163^+^ and CD163^-^ populations were very similar in terms of forward scatter (FSC) and side scatter (SSC) (Fig. 2c). CD80, CD86, CD1b, and CD40 were not included on the same analysis panel as CD163 and therefore differential expression of these markers was not assessed. Further analysis of CD172a (Fig. 3a) showed large differences in expression between the CD163^-^ and CD163^+^ subsets. CD172a versus ADGRE1 staining was assessed on both CD163^−^ and CD163^+^ subsets (Fig. 3b), and the CD163^+^ subset demonstrated greater expression of both CD172a and ADGRE1 in comparison to the CD163^-^ subset. In the CD163^-^ subset, a small ADGRE1^-^ population was observed, predicted to be lymphocytes (Fig. 3b).

**Table 3:** Mean (± SD) change in MFI for a range of molecules on CD163^+^ and CD163^-^ BAL cells.

	CD163 +		CD163-		*p*-value
	Mean change in MFI	SD	Mean change in MFI	SD	
CD163	6230.3	1417.9	201.0	93.7	0.004**
CD172a	4307.0	2242.0	1431.5	848.6	0.027*
ADGRE1	4487.5	1328.8	1982.0	525.8	0.010*
CD206	3481.3	819.8	1129.5	226.8	0.005*
CD14	2086.3	415.9	326.0	158.4	0.001**
MHCII	710.3	44.4	322.0	42.4	< 0.001***
CX3CR1	545.3	139.8	135.0	27.2	0.013*
CD16	265.5	145.7	70.0	84.1	0.019*
CD26	108.0	52.0	− 83.3	56.6	0.032*

*Denotes *p* < 0.05, ** denotes *p* < 0.01, and *** denotes *p* < 0.001 statistically significant difference between molecule expression on CD163^+^ and CD163^-^ BAL cells by two-tailed paired Students’ T-test. n = 4 biological replicates.

### Subsets of BAL myeloid cells differentially expressed CD163 and CD14

**Figure 4:**
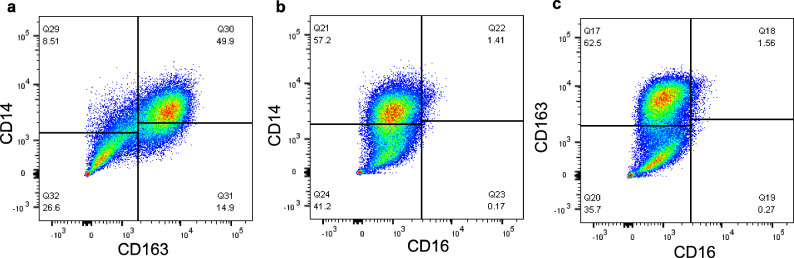
Two major populations of BAL cells are distinguished on the basis of CD163 and CD14 expression. BAL cells were stained for expression of a range of cell surface molecules and assessed by flow cytometry. Live, single cells were gated as shown in Supplementary Fig. 1 and gates were set as described in Supplementary Fig. 2. (**a**) CD163 vs CD14, (**b**) CD16 vs CD14, (**c**) CD16 vs CD163. One representative example of n = 4 biological replicates.

To determine the presence of classical and non-classical myeloid cell populations, expression of CD14, CD16 and CD163 on BAL was investigated further (Fig. 4). When cells were examined for co-expression of CD163 and CD14, there were two subsets of CD163^+^CD14^+^ cells (*Mean* = 39.1%, *SD* = 9.1%) and CD163^−^CD14^−^ cells (*Mean* = 34.5%, *SD* = 8.0%) (Fig. 4a) observed. As previously indicated in Fig. 1k, little or no CD16 expression was observed (Fig. 4b, c).

## Discussion

Bovine AMs are the first line of defence against inhaled pathogens such as *M. bovis*, however, little is known about the phenotype and heterogeneity of bovine AMs. This study describes the cell surface phenotype of bovine AMs, including the expression of two molecules for which we have recently generated reagents: ADGRE1 and CX3CR1. As described in porcine AMs^6^, ADGRE1 was expressed on the vast majority of bovine AMs at a high level. Conversely, expression of CX3CR1 by bovine AMs was uniformly low.

Although F4/80 is expressed by many murine macrophage types, there is very little expression by murine AMs^4^. Conversely, we found that bovine AMs have uniformly high expression of ADGRE1, comparable to porcine AMs^6^. Although studies in F4/80 deficient mice suggest that ADGRE1 may be involved in immune tolerance^19^, in pigs, the rapid evolution of this molecule and related ADGRE family members suggested immune selection and a role in pathogen recognition^6^. Further studies are required to determine roles for ADGRE1 in cattle.

We demonstrated here that the bovine AM population consists of distinct CD163^+^ and CD163^-^ subsets. Similarly, human AMs can be divided into CD163 high and CD163 intermediate subsets^11^. It has been suggested that the diversity in CD163 expression in human AMs may be an indication of whether the cells have foetal monocyte origins, or have been derived from circulating monocytes. It is also possible that CD163 expression correlates to the activation state or where the macrophage is localised within the lung^11^. Unlike human and bovine AMs, pig AMs have been shown to be uniformly CD163^+^^20^.

CD163 is a scavenger receptor exclusively expressed by monocytes and macrophages, and it has been used as a marker for anti-inflammatory M2 macrophages. To our knowledge, the functions of CD163 in bovine AMs have not been examined. Further investigations are required to determine potential roles in bovine health and disease. In our study, high expression of CD163 corresponded with increased expression of other macrophage markers in comparison to the CD163^−^ subset. Interestingly, MHC II was the molecule with the most statistically significant difference in expression between the CD163^+^ and CD163^-^ subsets. This suggests that the CD163^+^ subset may be more efficient at antigen presentation than the CD163^−^ cells.

Traditionally, macrophages have been classed as either belonging to the pro-inflammatory M1 class or the anti-inflammatory M2 class. In mouse and humans, MHC II is considered an M1 marker, whereas CD163 is considered an M2 marker. However, the appropriateness of the M1/M2 categorisation for AMs has been questioned as it has been shown that most human AMs have high expression levels of both M1 and M2 markers^21^. The vast majority of human AMs were categorised as CD206^hi^CD86^hi^, and this population also had higher levels of CD163, CD80, CD64 and HLADR compared to the other subsets. It has been suggested that this mixture of M1 and M2 features may allow AMs to maintain a balance between immunological protection and tolerance^21^. Whilst the M1/M2 categorisation has been studied extensively in humans and mice, macrophage polarisation in cattle has been less explored. In this study, we showed that bovine AMs express a mixture of molecules categorised as M1 and M2 markers. There were high expression levels of the M2 marker, CD206, and moderate expression levels of the M1 markers, CD80, MHC II, and CD40. Further studies of function will be required to elucidate functional roles for bovine AM subsets and determine whether they can be classified according to the M1/M2 paradigm.

Tissue resident AMs originate from foetal monocytes that colonise the lungs soon after birth. Whilst these AMs are capable of self-renewal, they can also be replenished by circulating monocytes. Therefore, in this study we analysed the expression of markers expressed by monocytes. It has been shown that bovine monocytes, like human monocytes, can be divided into three groups based upon the expression of CD14, CD16, and CD163. The majority of monocytes in the peripheral blood of cattle are classical monocytes (CD14^+^CD16^−^ CD163^hi^). There are also intermediate monocytes (CD14^+^CD16^+^) and non-classical monocytes (CD14^−^CD16^+^CD163^lo^)^9,22^. In this study it was shown that bovine AMs can be divided into two populations of CD163^+^CD14^+^ cells and CD163^-^CD14^−^ cells, both of which were CD16^−^. The CD163^+^CD14^+^ population, therefore, resembles the classical monocyte population.

It is likely that the three bovine monocyte subsets have different functions, however, different studies have yielded contradictory results due to differences in isolation techniques and gating strategies. For example, one study demonstrated that non-classical bovine monocytes are not inflammatory as their ability to phagocytose material, generate reactive oxygen species (ROS), and express LPS-induced IL-1β is reduced compared to the other subsets^22^. However, another study showed that non-classical monocytes had a greater endocytic capability compared to the other subsets, and expressed high levels of IL-1β upon stimulation^9^. Bovine monocyte subsets were shown to have different expression levels of chemokine receptors and antigen presentation and costimulatory molecules, indicating that they have different roles in detecting pathogens and processing antigens. For example, it was shown that classical monocytes have greater CD86 expression but lower CD1b and CX3CR1 expression compared to non-classical monocytes^9^. In our study, bovine AMs were shown to have low CD86 and CX3CR1 expression, and moderate CD1b expression. Interestingly, CX3CR1 was expressed at greater levels in the CD163^+^ subset compared to the CD163^-^ subset. Although the CD163^+^ CD14^+^ subset would appear to correlate with the classical monocyte population, CX3CR1 expression is greater in non-classical monocytes compared to classical monocytes.

The analysis of AMs by flow cytometry was challenging due to the high autofluorescence of the cells which made it difficult to accurately apply gating boundaries to distinguish positive and negative populations. Additional analysis of the cell subsets will be required to determine functional capabilities of the subsets and expression of additional key molecules. The BAL samples used in this study were extracted from male Holstein–Friesian calves between the ages of 12–24 days. It has been reported that bovine AMs mature during the first 3–6 months of life, and during this maturation period there are fluctuations in the number of CD14^+^ cells and the efficiency with which they carry out phagocytosis and generate ROS^23^. Therefore, expression of some molecules by AMs may differ in neonates and adult cattle, requiring additional study particularly in the context of chronic diseases such as bovine tuberculosis.

Here we have extended the phenotypic analysis of bovine AMs and have described the presence of two major subsets with differential CD163 expression. This characterisation of uninfected bovine AMs is essential for future studies which will examine the role of AMs in *M. bovis* infection.

### Supplementary Information

Supplementary Figures.

## Data Availability

Data supporting the findings of the study are available within the paper and its supplementary information.
