# Supplementary material for: Phenotypic characterisation of bovine alveolar macrophages reveals two major subsets with differential expression of CD163
Source: Sci Rep. 2024 Jun 28;14:14974. doi: 10.1038/s41598-024-65868-7 (PMC11217380; doi:10.1038/s41598-024-65868-7)
Supplement: Supplementary file 1 — Supplementary Figures. [file 41598_2024_65868_MOESM1_ESM.pdf]

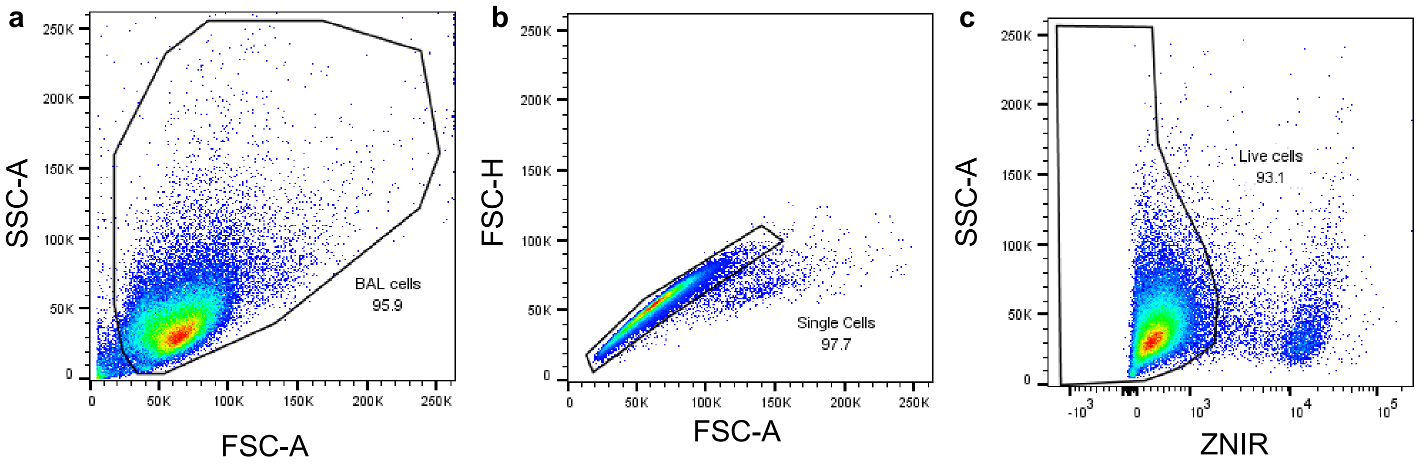

Supplementary Figure 1: Gating strategy for BAL flow cytometry analysis. BAL cells were washed and stained for a range of cell surface molecules. Cells were acquired using a BD LSRFortessa flow cytometer and analysed using FlowJo v10 software. The following gating strategy was used: **(a)** Debris was excluded using an FSC-A vs SSC-A gate. **(b)** Single cells were selected using an FSC-A vs FSC-H gate. **(c)** Live cells were selected using Zombie NIR viability dye staining.

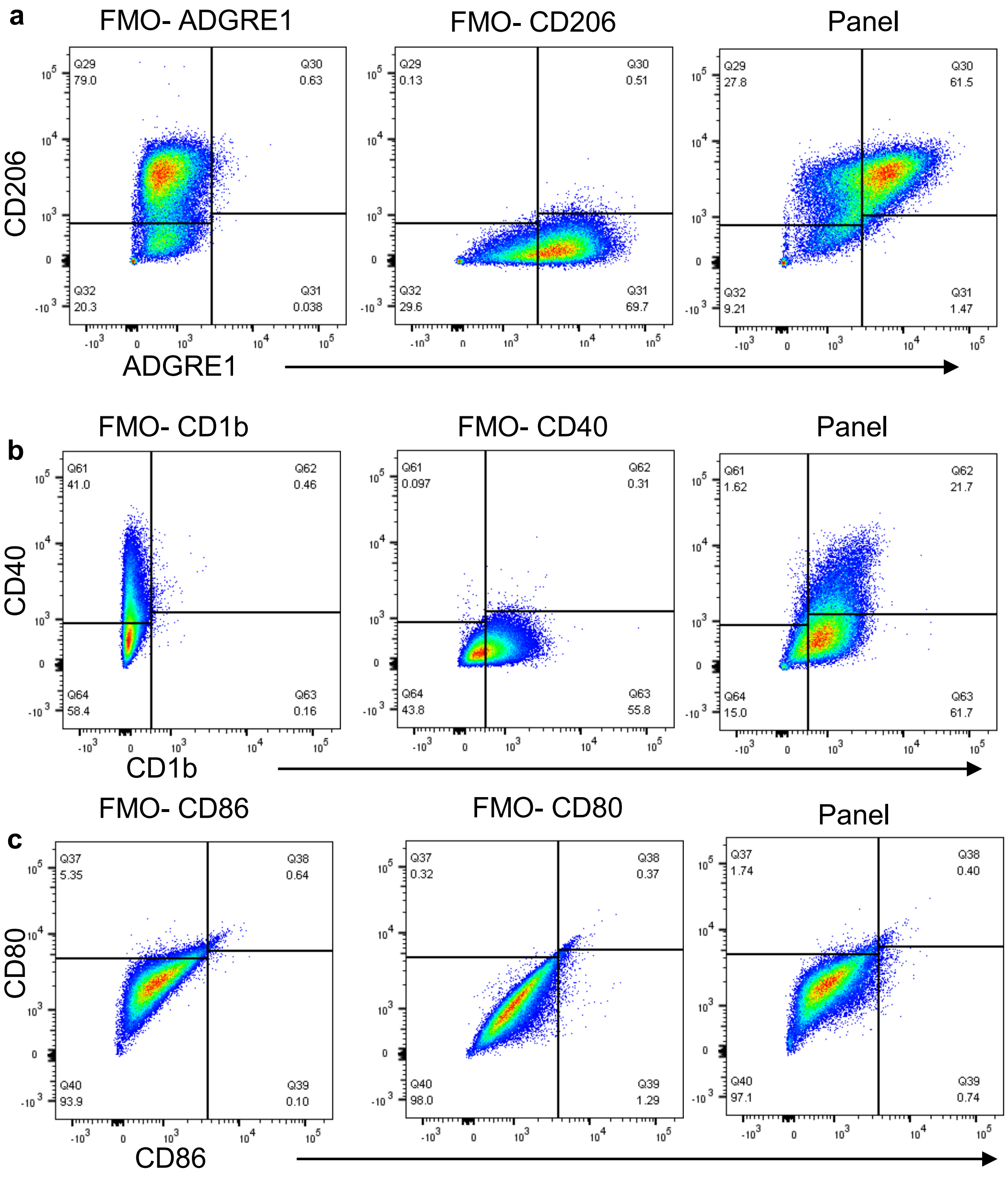

Supplementary Figure 2: Gating strategy using Fluorescence Minus One (FMO) controls. Cells were stained and acquired as shown in Supplementary Fig. 1. Gating was applied to approximate the proportion of cells which were single positive, double positive, and double negative for each combination of markers. Upper gate boundaries were set using the FMO controls. **(a)** ADGRE1 vs CD206 gating, **(b)** CD1b vs CD40 gating, **(c)** CD86 vs CD80 gating.
